# Big data, open science and the brain: lessons learned from genomics

**DOI:** 10.3389/fnhum.2014.00239

**Published:** 2014-05-16

**Authors:** Suparna Choudhury, Jennifer R. Fishman, Michelle L. McGowan, Eric T. Juengst

**Affiliations:** ^1^Division of Social and Transcultural Psychiatry, McGill University and Lady Davis Institute, Jewish General HospitalMontreal, QC, Canada; ^2^Biomedical Ethics Unit, Social Studies of Medicine Department, McGill UniversityMontreal, QC, Canada; ^3^Department of Bioethics, Case Western Reserve University School of MedicineCleveland, Ohio, USA; ^4^Center for Bioethics, University of North CarolinaChapel Hill, NC, USA

**Keywords:** open neuroscience, open science, data sharing, neuroimaging, human genome project, brain initiative, human brain project

## Abstract

The BRAIN Initiative aims to break new ground in the scale and speed of data collection in neuroscience, requiring tools to handle data in the magnitude of yottabytes (10^24^). The scale, investment and organization of it are being compared to the Human Genome Project (HGP), which has exemplified “big science” for biology. In line with the trend towards Big Data in genomic research, the promise of the BRAIN Initiative, as well as the European Human Brain Project, rests on the possibility to amass vast quantities of data to model the complex interactions between the brain and behavior and inform the diagnosis and prevention of neurological disorders and psychiatric disease. Advocates of this “data driven” paradigm in neuroscience argue that harnessing the large quantities of data generated across laboratories worldwide has numerous methodological, ethical and economic advantages, but it requires the neuroscience community to adopt a culture of data sharing and open access to benefit from them. In this article, we examine the rationale for data sharing among advocates and briefly exemplify these in terms of new “open neuroscience” projects. Then, drawing on the frequently invoked model of data sharing in genomics, we go on to demonstrate the complexities of data sharing, shedding light on the sociological and ethical challenges within the realms of institutions, researchers and participants, namely dilemmas around public/private interests in data, (lack of) motivation to share in the academic community, and potential loss of participant anonymity. Our paper serves to highlight some foreseeable tensions around data sharing relevant to the emergent “open neuroscience” movement.

## Introduction

Echoing the ambitions of George H. W. Bush’s Decade of the Brain and the National Institute of Mental Health (NIMH)’s recent statement that it will be reorienting its research towards a new taxonomy based on brain structure and function (Insel and Lieberman, 2013), the Obama Administration’s announcement of the Brain Research Through Advancing Innovative Neurotechnologies (BRAIN) Initiative in 2013 reflects the continued hope and investment in neuroscience research for understanding human brain structure and function, in particular for its applications in psychiatry. BRAIN’s challenge, according to the Interim Report compiled by NIH, “is to map the circuits of the brain, measure the fluctuating patterns of electrical and chemical activity flowing within those circuits, and understand how their interplay creates our unique cognitive and behavioral capabilities” (Advisory Committee to the Director, 2013, p. 8). Funded largely by the Defense Advanced Research Projects Agency (DARPA), National Institutes of Health (NIH) and private research institutes including the Allen Institute for Brain Science and the Kavli Foundation, the BRAIN Initiative is set to rival the European Commission’s $1.3 billion Human Brain Project, which, according to the project’s director, will be the “Higgs boson of the brain” (Honigsbaum, 2013). Indeed, both projects mark bold efforts to accelerate the effort to map the human brain, and both have been repeatedly compared with the Human Genome Project (HGP) in terms of the value of mapping the brain’s intricate web of connections, the “connectome” (Kaye et al., 2009; Milham, 2012; Leonelli, 2014). Analogous to the genome—and the HGP’s symbolic meaning of sequencing three billion nucleotides that represent human inheritance—the goal to map the brain’s networks is a feat understood to be no less than mapping human identity and “the wiring that makes us who we are” (Seung, 2012).

While there is certainly a growing level of scepticism and ethical scrutiny in relation to the “neurologization” of understandings of mental health, selfhood and notions of human nature based on incomplete findings from the field (e.g., Racine et al., 2005, 2010; Illes et al., 2010; Choudhury and Slaby, 2011; Pickersgill, 2011; Vidal and Ortega, 2012; Rose and Abi-Rached, 2013), there is no doubt that the last 25 years have seen extraordinary conceptual and technological growth and development in the human neurosciences enabling the production of enormous quantities of brain data. Explanatory trends in these subfields have begun to move beyond universalistic models to investigate inter-individual differences, interactional dynamics between people, and between individuals and their environments over time. Cognitive neuroscience research is thus increasingly characterized by the goal to understand the relationship between functional brain organization and behavior by analyzing covariance in large-scale studies. Converging with the goals of epigenetics and genomics research, the focus is shifting toward understanding how inter-individual differences are shaped by an interaction of genetics, the brain and experience, and how these mechanisms influence normal behavior and susceptibility for mental disorders. In line with these goals, neuroscientists have begun to use discovery-based approaches to facilitate statistically robust investigation of brain–behavior relationships, through the culling of large-scale data sets. To quote Michael Milham, an advocate of “open neuroscience”, “human neuroimaging has entered the connectome-wide association (CWA) era. As with genome-wide association studies (GWAS) the objective is clear: to attribute phenotypic variation among individuals to differences in the macro- and microarchitecture of the human connectome” (Milham, 2012, p. 214).

Through their scale, paradigms and organizational structures, the BRAIN Initiative and the Human Brain Project exemplify a shift in the field towards high-powered data-driven research, and the corresponding move towards large-scale data-sharing. BRAIN’s Interim report states that the “organization and mining of “big data” sets can radically accelerate” (Advisory Committee to the Director, 2013, p. 10) understanding of the relationship between neuronal activity and behavior. To “understand the secrets embedded in [the] data” (p. 13) the report, consistent with other emerging initiatives within neuroscience, underscores the importance of collaboration between laboratories around the world in order to pool, store and harness unprecedented quantities of brain data. In this article we focus specifically on data sharing as a particular point of emphasis and comparison drawn between the BRAIN Initiative and the HGP and its outgrowths. We begin by describing the emerging “open neuroscience” movement, demonstrating the reasons, and ways in which, neuroscientists are encouraged to contribute to large open access archives of neuroimaging data. We then look at case studies from genomic research to examine the complexities of data-sharing in order to draw lessons about the social and ethical challenges that may be relevant in the era of “open neuroscience” and new initiatives including BRAIN and the Human Brain Project.

## Big data, the brain and the impetus to share

“The age of “big data” for the brain is upon us. Thus, neuroscientists are seeking increasingly close collaborations with experts in computation, statistics and theory in order to mine and understand the secrets embedded in their data”.

*(Advisory Committee to the Director, 2013, p. 13)*

Calls to make the growing banks of brain data, analytic tools and protocols publicly and freely accessible have recently garnered increasing strength and visibility, pervading the texts released so far by the committee for the BRAIN Initiative (Advisory Committee to the Director, 2013, pp. 47–51) and other big data projects emergent in neuroscience (e.g., The Human Brain Project, 2012, p. 23). There are multiple reasons stated for this growing drive. First of all, neuroscience research yields enormous quantities of complex data at various levels of study and open access to data in shared repositories offers the potential to integrate, re-use and re-analyze data. Datasets from neuroimaging studies generally contain more information than one lab has the methodological and interpretive expertise to extract; data sharing therefore maximizes the utility of data and skills of researchers, accelerating the pace of investigations around particular questions (Poline et al., 2012; The Human Brain Project, 2012, p. 44; Poldrack et al., 2013). Furthermore, neuroimaging is a costly method; typically functional MRI (fMRI) experiments involve 10–15 participants at a cost of at least $300/hour for their scans (Poline et al., 2012). Studies generate large amounts of data (gigabytes) and while their findings are generally published, few are replicated in view of these costs as well as the culture of rapid publishing of novel results in neuroimaging research. Data-sharing not only affords much greater sample sizes and therefore better quality of data, correcting for effects of noise or other errors (Milham, 2012); it also becomes an economic imperative at a moment in which funding institutions and universities have limited resources. Data sharing, the advocates argue, is therefore a crucial imperative from a scientific point of view—to increase statistical rigor and open up interpretive possibilities (Nature Neuroscience, 2000; Gardner et al., 2008; Poldrack et al., 2013), and to step up the pace of research, realizing its translational potential for medicine (Poline et al., 2012; The Human Brain Project, 2012, p. 55).

Alongside the acceptance of the scientific importance of data sharing in biology, open data sharing has also become a matter of professional moral obligation between scientists. Scientific secrecy, once defended by notions of academic freedom, scientific integrity, and intellectual competition, is now considered a professional vice amongst scientists, warned against in training programs alongside other forms of scientific misconduct like data fabrication and plagiarism (MacFarlane, 2008). This is equally true in the neurosciences, where the calls for open access to data have followed a succession of controversies in the neuroimaging community regarding statistical shortcomings of certain findings and limited reproducibility of others, owing in part to the plurality of data analysis methods (Jabbi et al., 2009; Vul et al., 2009; Margulies, 2011; Carp, 2012; Poline et al., 2012). Data sharing therefore responds to the increasing call within the scientific community and within the public at large for greater access to raw data and general transparency (Visscher and Weissman, 2011). Furthermore, while advocates of open neuroscience tend to focus their arguments on the methodological benefits, some neuroscientists believe that data sharing is a moral virtue that should be incorporated into the normal practices of all neuroscientists. These researchers argue that data sharing is an ethical duty of researchers to fulfil their obligations to research participants, by fully respecting and maximizing their contributions (Brakewood and Poldrack, 2013). They urge that recognition of these benefits and duties is necessary to initiate wide scale cultural reform within the neuroscience community and to foster a spirit of collaboration across laboratories (Milham, 2012).

As a result of these professional ethical and scientific imperatives, the BRAIN Interim Report stresses the need to create the appropriate infrastructural arrangements to establish data-sharing platforms. Recognizing the loss of vast quantities of data, “siloed” in their originating labs, the report promotes the design and establishment of well-curated data platforms enabling easy access to data as well as standardized analytic tools (Advisory Committee to the Director, 2013, pp. 50–51). Similarly, the report by the Human Brain Project’s Consortium states that this “potentially revolutionary change in current research practices” (The Human Brain Project, 2012, p. 55) will lead to greater efficiency in data use as well as enable integration of data from studies of different levels of brain organization. While this call to open up access to data among neuroscientists is not new, the urgency to develop the means to do so is increasing, as evidenced by mounting pressure from funding bodies, academic presses and universities. One of the driving reasons for this is also the ever-growing datasets generated by new technologies, many of which are unstable owing to their sheer size.

Among researchers, the response to these calls to share data in neuroscience has been slow. Long before the announcement of the BRAIN Initiative or the Human Brain Project, cognitive neuroscientist and Director of the SAGE Center for the Study of the Mind, Michael Gazzaniga famously brought attention to these issues when he established a public archive at Dartmouth College for fMRI data to be deposited and openly accessed. As editor of the *Journal of Cognitive Neuroscience*, he also initiated a journal requirement that authors release their data with publication of their papers. These steps were controversial and met with considerable levels of scepticism by the neuroscience community, the reasons for which we return to below in the next section (Nature, 2000). However, since 2000, there have been significant shifts among researchers to promote and enable sharing of fMRI data (Van Horn and Gazzaniga, 2013) through the establishment of organizations such as the International Neuroinformatics Coordinating Facility (INCF),“bottom-up” initiatives to instantiate open access archives (Milham, 2012), and not least, the forthcoming launch of *Scientific Data*, an online publication for data descriptions, re-use and re-analysis^1^ by the Nature Publishing Group. In the following section, we outline the goals of some of these new data repositories emerging under the name of “open neuroscience”.

## Emerging practices of data sharing in cognitive neuroscience

A handful of initiatives for sharing analytic tools and data have existed for about a decade (e.g., EEGLAB’s open source toolbox (Delorme and Makeig, 2004) and the Neuroscience Information Framework (Gardner et al., 2008)). However, the sharing of neuroimaging data has only very recently gained momentum through the “open neuroscience” movement, which has become institutionalized through academic publications, a website, the formation of informal and formal networks and spaces for collaboration across disciplines and the sharing of neuroinformatics tools and data.^2^

The subfield of resting-state fMRI (rsfMRI), in particular, illustrates an area of research that has demonstrated the benefits of such data sharing. The field enables the study of the correlates of a range of behavioral processes through investigations of “functional connectivity” based in the correlation of spontaneous brain activity (Biswal et al., 1995). Resting-state paradigms offer a valuable methodological advantage, with potential applications for clinical research, in view of the relatively higher samples possible with fewer costs relative to PET and traditional task-based fMRI studies. Resting-state studies allow data to be openly shared and publicly distributed on the order of hundreds of patients and matched healthy control data sets, culled across multiple institutions. Such availability of data facilitates cross-site validation and appropriate statistical power for addressing complex brain–behavior relationships that are especially necessary for clinical populations. Resting-state fMRI data have also been shown to have high reproducibility and test-retest reliability (Milham, 2012).

Eyeing these advantages for psychiatric neuroimaging, new data consortia have developed under the umbrella of open neuroscience (Milham, 2012) with a view to aggregating data from multiple studies in order to generate clinically useful predictive models including the detection of image-based biomarkers. The results have produced successful models for the potential of data sharing to enable the gathering of large data sets, perform new analyses, and generate new testable hypotheses. For example, the 1000 Functional Connectomes group enabled the release of a huge data set from over 1000 participants across 30 sites (Milham, 2012). As part of this group’s initiatives, the ADHD-200 Global Competition^3^ provided an impetus for several labs to make their data available in an effort towards the development of predictive tools for ADHD diagnosis using resting-state data. In a similar vein, the Functional Biomedical Informatics Research Network (FBIRN) promoted the sharing of data related to schizophrenia (Glover et al., 2012) which has enabled new analyses drawing on data from multiple sites to point to novel findings about memory (Kim et al., 2009; Potkin et al., 2009). Resting-state fMRI data archived in these repositories became the basis for capturing phenotypic diversity in brain–behavior relationships (Kelly et al., 2012), and for challenging existing psychiatric classifications by performing powerful statistical tests of the probability that specific functional connectivity relationships covary with any phenotypic measure-of-interest such as personality (Adelstein et al., 2011; Hahn et al., 2012; Wei et al., 2014) or social behavior (Di Martino et al., 2009; Cox et al., 2012). Following this model, there have also been recent calls to share data from task-based fMRI paradigms through the Open fMRI project (Poldrack et al., 2013) to generate higher quality multivariate analyses of relationships between cognitive processes and brain function. Similarly, projects like Brainmap,^4^ the Open Access Series of Imaging Studies (OASIS)^5^ and Neurosynth^6^ provide tools for the research community to access MRI and fMRI data to enable meta-analysis of clinical and non-clinical populations, while the growing Human Connectome Project^7^ enables sharing of data from studies using multiple MR modalities including diffusion imaging, resting state fMRI and magnetoencephalography (MEG) with the goal of mapping human brain connectivity as accurately as possible.

## Challenges to data sharing: looking to genomics

Despite the professional ethics of data sharing and the many methodological benefits, the culture of research has been slow to shift towards open neuroscience and most imaging data remains inaccessible. A recent survey about data-sharing practices among scientists revealed considerable unwillingness to disclose whether or not they share data. Nearly half of the respondents said they do not share data, citing reasons of lack of time, underdeveloped standards, and inadequate infrastructure. Interestingly, 85% of these respondents indicated an interest in having access to other researchers’ datasets (Tenopir et al., 2011). The gap between the motivation to share data, and the desire to use available data sets raises interesting questions. Researchers have begun to identify barriers to data-sharing, specifically within neuroscience, and have identified technical and infrastructural difficulties that exist which require strong motivation among researchers to spend the time and effort in learning, for example, ways in which to effectively share, aggregate and archive their data (Milham, 2012; Poline et al., 2012). Consensus on issues of appropriate descriptors to accompany raw or processed data, the means to move data and the format of it remains to be resolved in the neuroimaging community.

However, our focus is not on technical barriers, because the most significant challenges to data sharing in this field are sociological and ethical. In the neuroscience community specifically, individual researchers’ lack of motivation to share is considered a key obstacle to wider change in data-sharing practices (Poline and Poldrack, 2013). In particular, a major barrier is the competition to be the first to analyze data, and to be recognized for novel findings. In an academic context in which funding is increasingly competitive, and data are relatively expensive to generate, anxieties about being “scooped”, or undercut, by other data collectors constitute a very real challenge to the cultural reform envisaged by open neuroscience advocates. Moreover, neuroscientists may also be concerned about the quality of the data and fear being scrutinized publicly for inadequate paradigms or data collection methods, particularly after the very public forms of criticism of neuroimaging analysis mentioned earlier, which initially used freely accessible online forums for criticism rather than peer reviewed academic journals (Vul et al., 2009; Margulies, 2011).

Researchers’ willingness to share data can also be constrained by concerns for the privacy of the human research participants who are the data sources, and the data-sharing permissions they have granted in consenting to participate (Van Horn and Gazzaniga, 2013). Currently, most informed consent forms completed by participants for neuroimaging studies cover the consent for the use of the participant’s data for the research questions related to the primary study focus and not for potentially unrelated investigations that could follow from open access to these data in the wider community. Although efforts are underway to develop widely-shared policies, as evidenced by efforts among research groups involved in the Databrary Project^8^ and the Human Connectome Project,^9^ regulatory mechanisms for consent for use of data in the context of open access databases have not been fully worked out. As has been discussed in the field of genomics (McEwen et al., 2013), there is further concern in neuroscience that wide scale use of brain imaging data opens up the possibilities for re-identifiability of participants. Neuroimaging data coupled with layers of descriptive meta-data may mean that “sulcal and gyral fingerprints” (Poline et al., 2012, p. 6) or even BOLD activity patterns could compromise participant confidentiality, even when the data has been “anonymized” in ordinary ways. Furthermore, the heavy focus on public-private partnerships involved in funding big data projects for the BRAIN Initiative leave open numerous questions about the applications of these data, and the tensions that may exist between public and private interests and the forms of “benefit-sharing” participants who contribute their data might expect. Discussion of these ethical issues as they pertain to data sharing in cognitive neuroscience remains highly limited at present. They have been extensively studied in the genomics research context, however, and review of that scholarship suggests that sociological and ethical issues are essential to understanding and confronting the limits and resistance to open data.

Advocates of open neuroscience frequently invoke the success of research in genomics as a model for data sharing, citing the GenBank and Hapmap archives as examples that led to important genetic discoveries (Manolio et al., 2008; Poldrack et al., 2013; Van Horn and Gazzaniga, 2013). Efforts among organizations such as the INCF to promote collaboration and sharing have been compared to the guidelines established in the genomics community that set a precedent for the creation of data platforms, and more importantly, research cultures, that foster successful data sharing. Although genomics is heralded as a relative success story in the realm of openness, researchers have also demonstrated that the shift towards data sharing was not as seamless as it is frequently described (Jasny, 2013). Case studies, such as the multisite eMERGE Consortium for GWAS, have revealed that in reality, institutions involved in data sharing face several challenges in terms of bureaucracy and infrastructure (McGuire et al., 2011). Studies have also shown that hindrances to data sharing in genomics arise as a result of researcher dilemmas around credit sharing in the academic economy (Blumenthal et al., 2006; McGuire et al., 2011; Nanda and Kowalczuk, 2014) as well as ambiguity about ethical standards to protect research participants. In the following section, we further explore these challenges to genomic data sharing and attempts to overcome such obstacles.

## The Politics of Openness: the context of genomics

### Public/private interests in genomic data

Recognizing significant interest from both public and private entities in achieving its goals, promoters of the HGP argued that sequencing the human genome would be greatly accelerated through collaboration and sharing of technological and financial resources. A coordinated public/private partnership involving the United States’ NIH and Department of Energy, The Wellcome Trust, and the private corporation of Celera was proposed to generate a draft sequence of the human genome using composites of 17 individuals. The hopes were that this partnership would reduce duplicative efforts and allow both private industry and public scientists to reap the rewards of efforts to sequence the genome with open access to data deposited in the GenBank public repository, though with some intellectual property rights in the data retained (Jasny, 2013). Despite a public face of coordinated effort, in reality the race to sequence the human genome was more like a competition between public and private interests in which neither side achieved their goals of a clean and complete publicly available sequence or a profitable private sequence in which all users would pay to view the results (Jasny, 2013). The challenges faced by the public/private partnership of the HGP suggest that there may be some incompatibility in the goals of these types of organizations when they endeavor to share large-scale data.

Following the completion of the HGP, the President’s Council of Advisors on Science and Technology (PCAST) (2008) recommended the development of a strategic long-term plan to streamline the coordination of public and private efforts to develop tools and technologies to forward genomic research and medicine. Pointing to the historical separation of discovery research in the publicly-funded research sector and research validation in the private sector, PCAST was particularly concerned with coordinating public and private efforts to validate genetic disease correlations that would allow genomic research to be successfully translated into clinical applications. The solutions proposed by this federal body included: (1) increased public investment in translational research to complement industry-sponsored efforts; and (2) federal leadership and funding for integrated public/private biorepositories to support genomic research and academic/private collaborative research projects. Since these initial endeavors to bring together public and private interests in genomic data around shared scientific goals, several policy efforts have been initiated to promote genomic sample and data sharing, including the SNP Consortium, the HAPMAP, eMERGE, ENGAGE, and H3Africa (McEwen et al., 2013). Despite increased attention to arrangements that mutually benefit public and private interests, these efforts have encountered challenges to data-sharing pertaining to participant community values and national claims to ownership and control of genomic data under the concept of “genomic sovereignty”, suggesting that participant perspectives on data-sharing received insufficient attention in early genomic data-sharing strategies (de Vries and Pepper, 2012).

### Openness/Secrecy: professional dilemmas about sharing

In order to “maximi[ze] the scientific yield from research data collections”, funders of genomic research now often require data sharing across research teams and consortia (Budin-Ljøsne et al., 2014, p. 1). Further, data sharing is seen as essential for the conduct of cutting-edge genomic research. As a result, the genomic sciences have instituted infrastructural conduits and safeguards to encourage openness. Despite these accommodations, genomic scientists, their academic institutions, and the wider community continue to face sociological and ethical challenges alongside recalcitrant professional norms that impede realization of the promise of data sharing.

Over the past 10 years, numerous strong, collegial collaborations have been formed in order to promote data sharing. In particular, these collaborative, trans-institutional organizations have instituted mechanisms to encourage sharing while protecting the integrity of the data. For example, data storage and management is often handled by “honest brokers”, utilizing centralized systems that control access to the data and have concomitant requirements about depositing aggregate study results for use by many (Jeffers, 2001; Winickoff and Winickoff, 2003; Yassin et al., 2010). A further safeguard is to have separate repositories for summary level data and individual data, the latter of which is kept under restricted access, as is specified by NIH’s GWAS data access policy (Kaye, 2011; McEwen et al., 2013). The same is true for the HapMap project which uses Coriell’s repository, which has specific access rules and regulations. Often access to data is controlled by committees who must determine whether uses of the data are appropriate, ethical, and follow policy guidelines (McEwen et al., 2013).

These safeguards, while necessary, can also act as impediments to data access. In addition to the bottlenecks caused by slow data harmonization within and across consortia, it is also difficult to share data beyond pre-established consortia members due to lack of standardization of data-sharing policies (Budin-Ljøsne et al., 2014). Decisions made by committee can be laborious and time consuming, delaying research. Given that many of these repositories are developed through public-private partnerships, different policies and norms around data ownership can delay or forestall collaborative research even further, despite the best of intentions towards openness (Jasny, 2013).

A further challenge for academics who may wish to shift towards more open models in their research is the professional norms around credit sharing in the academic economy (Campbell et al., 2002; Blumenthal et al., 2006). While it may be in the interest of scientific progress as well as changing professional scientific norms (MacFarlane, 2008) to share data and work in an “open” model, academics still confront an economy where credit is given based on authorship status on publications. Publications by whole consortia or with numerous authors still present challenges for academics concerned about how these publications will be credited and recognized by their institutions (Blumenthal et al., 2006). Academia has, thus far, been slow to respond to these changes in the organization of scientific research such that credit can be attributed adequately for large team-based research. As a result, researchers have lacked motivation and incentives to contribute to data-sharing networks. While contributors have been acknowledged in publications under the umbrella name of their research team, such as HAPMAP (e.g., International HapMap Consortium, 2003), along with several in a long list of authors, the recognition of this kind of authorship by universities is currently uneven, even though such publications are widely cited.

### Anonymity and Reidentifiability of Research Participants

A third major challenge to open data sharing that genomics foreshadows for neuroscience flows from the fact that, unlike data sharing in physics or open source programming in computer science, data about human brains comes from people. This means that, beyond whatever personal and structural barriers to data sharing may exist, conscientious researchers must also ensure that they respect their subjects’ rights to control their participation in research, and protect the confidentiality of any data that can be traced to its human source. Traditionally, the former obligation has been discharged by restricting research to studies discussed with the participant during the informed consent process, and re-consenting participants for any new research conducted with their identifiable samples or data. For projects in which biospecimens or biomedical data are explicitly donated by participants with “broad consent” for unspecified future uses via biorepositories or shared data-bases, the traditional approach has been to “anonymize” the samples or data by severing all links between the data and its human sources (Haga and O’Daniel, 2011; McEwen et al., 2013). This has been understood to decrease the professional ethical imperative for specific consent by eliminating any downstream risks to the participants, and to effectively protect the confidentiality of their contributions. For research which has the potential to generate important clinical information about individual participants, arrangements are sometimes made to allow some party in the process, such as the biobank or data-base manager, to keep the key to re-identifying data sources should the need arise, under stringent privacy protections (McCarty et al., 2008).

In genomics, problems have emerged to challenge this traditional approach, each of which may also arise in the neuroscience context. First, as the genomic data about individuals that is available from open databases becomes increasingly comprehensive and cross-linked to the other forms of clinical, environmental, and genealogical information critical for specific genomic studies, it has proven possible to “re-identify” specific individuals as participants in genomic research (Gymrek et al., 2013; Williams, 2013). This immediately undermines the privacy of the information, raising important confidentiality concerns for scientists. But even more importantly, the ability to re-identify individual participants also raises the participants’ stake in controlling the kinds of research conducted with their samples and data, undercutting the ability of “broad consent” to provide adequate warrants for open data sharing.

A second challenge to traditional approaches to protecting participant interests in genomic research is the fact that, even where individual identities can be safeguarded, the prospect for group harms remains. A central strategy in genomic research is to compare the genomic profiles of different human groups in order to identify the variants that explain their phenotypic differences. Since these groups are usually defined by criteria that also have important social functions—i.e., by geographical boundaries, race/ethnicity, SES, or genealogical ties—they can be more sensitive to the social risks—and benefits—of scientific generalizations than individual participants. As a result, families, communities and national governments have begun to assert claims to “genomic sovereignty” over samples and data from members of their populations, introducing powerful political, economic, and legal complications for scientists who might otherwise be willing to share their data openly (e.g., de Vries and Pepper, 2012).

One important ingredient in the genomic debates over these challenges to wide data sharing is uncertainty over the actual social risks of genomic research results, either for individuals or groups. Much remains to be done towards assessing and quantifying risks to privacy that may result from data sharing (Clayton et al., 2010; Craig et al., 2011; Haga and O’Daniel, 2011) and in determining the effectiveness of public policy protections already in place, such as the Genetic Information Non-Discrimination Act (GINA) in the U.S. (McEwen et al., 2013; Robinson et al., 2013). Similar research challenges will be even more important for the neuroscientific community, since the causal links between the brain and all the human behaviors that trigger our social judgments are even more direct (and thus potentially more stigmatizing) than even the most deterministic genomic hypotheses can claim.

## Discussion

The debates within the neuroscience community, evident from weblogs, newspaper and magazine articles (e.g., Mitra, 2013; Requarth, 2013; Shen, 2013; Stein, 2013; Zwerdling, 2013), have reflected a degree of confusion and scepticism after the announcement of the BRAIN Initiative. Critics have argued that the goals have been poorly specified; that the parallels drawn with the HGP are tenuous in view of the lack of criteria for success or endpoints for the brain-mapping project. The controversy was intensified after Larry Swanson, president of the Society for Neuroscience, appealed to fellow neuroscientists to limit public criticism and debate in such a way that “our community be perceived as positive about the incredible opportunity” lest “[neuroscientists] are perceived as unreasonably negative or critical about initial details [and] … risk smothering the initiative before it gets started” (Swanson, 2013). Although potential ethical challenges have begun to be discussed,^10^ the controversy that has characterized the initial response to the announcement of the BRAIN Initiative—in terms of its precise objectives, its funding, and expected outcomes—indicate that several core issues remain to be resolved. We have argued that data sharing is among these issues that requires careful consideration.

As we have shown, the field of genomics does not provide a model of straightforward success in data sharing for biomedical research. However, its experiences and precedence can help the neuroscience community anticipate the challenges and complexities it is likely to face. Little is currently known about the extent to which the scientific goals of public and private investors in the BRAIN Initiative overlap, nor the kinds of data they expect to draw from projects they fund. It is not yet clear, for example, how DARPA’s objectives will be reconciled with the Allen Institute or Salk Institute’s research questions, much less what kinds of mechanisms, potentially analogous to those instituted in the case of genomics, will promote data sharing between them. Genomics research has also demonstrated that participant perspectives must be seriously considered throughout the process of developing brain data-sharing strategies as the Initiative evolves.

Experience from genomics has demonstrated that in order to motivate professional academics to deposit data into shared repositories, norms must be consensually set by committees who determine policy guidelines to facilitate the aggregation of data in standardized ways. Neuroscientists have already begun to follow suit: Open fMRI is a good example of a project that has established helpful standards and tools to enable easier data-sharing in the community. The organizers provide specifications and standards for data, which helpfully attempt to minimize barriers to data sharing in the community in order to facilitate whole brain meta-analyses (Poldrack et al., 2013). However, neuroscientists need to remain mindful about possible delays and bottlenecks to sharing caused by laborious guidelines, particularly when the norms and requirements of public and private sources may be in conflict. More importantly, granting agencies, universities and research institutes must address the crucial issue of academic credit, and devise methods that recognize and reward data sharing and encourage a culture of openness. This will include considerations about how best to reflect academic output and avenues for academic publication that encourage data acquisition and sharing as important contributions to the literature. It has been suggested that *h*-indices, metrics of publication citation, as measures of performance, are already a useful way to capture a result of data sharing, as long as a system is ensured for citing data from repositories that are used for analysis and re-analysis by authors other than the data generators (Poline et al., 2012). Acknowledging the dilemmas involved in data sharing among individual neuroscientists, particularly among junior investigators, Gorgolewski et al. (2013) have recently proposed guidelines for rewarding individual data generators through “data papers”, which, while common to other fields such as genetics, robotics, and earth sciences, are lacking in neuroscience. These data papers, which would serve to detail the experimental protocol and data specification without covering analysis or interpretation, might provide a mechanism for citable professional credit to the data generators. The authors suggest that data papers solve the problem of motivation for individuals to share data while “making it count” in the university system of merit, and at the same time allow different data users to draw on the same data sets for different interpretations, consistent with a central epistemological goal of open neuroscience (Gorgolewski et al., 2013).

Moreover, grassroots initiatives including research sites such as the Neurobureau, events like Brainhack and challenges like the ADHD-200 competition mentioned above, reflect an emerging commitment to an ethos of openness among young scholars who not only show motivation to share data but to develop the infrastructure to facilitate it, in a culture that fosters collaboration as well as transparency. Digital media and open-source software and databases have opened up the scope for establishing and sustaining the networks that enable large scale data sharing in these ways. Outgrowths from genomics research have shown that the commitment to openness, transparency, and translatability of research has led to new formations of research groups, established around values of participation and “citizen science” (Prainsack, 2014). Emerging projects and groups in neuroscience such as Eyewire,^11^ Backyard Brains^12^ or mcb80x.org reflect similar trends, and will likely lead to new challenges and possibilities for data sharing.

Finally, the realities of data sharing in neuroscience will include confronting issues of participant privacy that genomics researchers have struggled to manage. Neuroscience committees such as the INCF have plans to develop best practices and standardized ethics review for neuroimaging protocols that aim to respond to researchers’ anxieties about the lack of ethical guidelines for sharing participant data (Poline et al., 2012) and participants’ hesitation to contribute to experiments whose findings will be shared.

One approach to this challenge has been to call for research volunteers who are “information altruists” with respect to their biomedical data, willing to share fully identified personal genomic data for any and all research purposes (Kohane and Altman, 2005). Since empirical research suggests that many participants in genomic research may prefer restricted release of data (Haga and O’Daniel, 2011; McGuire et al., 2011; Oliver et al., 2012) and only those well-buffered from the social risks of exposing their future health vulnerabilities could afford to volunteer under this approach, it may not be capable of meeting neuroscience’s wider recruitment needs. To address this limitation in genomics, the same “honest broker” and “stewardship” models that are used to protect participant confidentiality are sometimes adapted to put proxy decision-makers such as Data Access Committees or Community Advisory Boards in place to police broad consent agreements on individual participants’ behalf, but not without continued ethical controversy (McCarty et al., 2008). To the extent that brain research could yield similarly unique neurological markers linking research findings with individual human beings, these challenges to data sharing are likely to arise in the neurosciences as well. Furthermore, as brain science increasingly adopts genomics’ comparative approach in attempting to identify the neurobiological bases of phenotypic variation across social, cultural and clinical groups, neuroscientists can expect to face similar political and economic challenges to their data-sharing ambitions as seen in the case of genomics.

In terms of the broader uncertainty surrounding the actual risks for individuals and groups that arise from sharing biological data, genomics researchers have attempted to limit fears among the public about the possibility to define personal identity, predict future traits or characterize human groups from genomic data by raising critical public understanding of the complexity of genomic regulation and the interactions between genes and environments (Nelkin and Lindee, 2004). Given that cognitive neuroscience, especially neuroimaging, has had analogous popular interest and popular press to genomics, riddled with metaphors about “mind-reading” capacities of neuroimaging and essentialistic hype about brain scans and personal identity, data-sharing advocates might consider stepping up critical public understanding of neuroscience research to emphasise the limits to the degree researchers can extrapolate from imaging data. Moreover, while neuroscientists have acknowledged that consent forms may need revisions to incorporate “anonymous” reuse of their data for applications beyond the immediate scope of the study, the degree to which anonymity can be preserved—when requirements for several levels of meta-data (clinical, environmental, genealogical) are required for the repositories—needs further discussion.

In light of the heavy emphasis on data-sharing initiatives that would facilitate the visions of archives of big data for future neuroscience, we suggest that the challenges associated with data-sharing practices need to be carefully examined. Genomics research—which frequently serves as the model for successful data sharing among open neuroscience advocates—highlights a number of important challenges that may be faced by neuroscientists. In particular, here we have singled out sociological and ethical challenges that have had limited attention in the neuroscience community thus far. Importantly, these examples demonstrate that the politics of openness are complex—presence of infrastructure and technical capabilities alone will not enable widespread data sharing. The cultural shift called for by open neuroscience advocates requires rigorous and open debate about the (potentially competing) goals of public and private investment in brain research, academic incentives to collaborate and share data in an increasingly competitive research context as well as standards that will protect the privacy of participants willing to contribute their data to experimental research.

## Conflict of interest statement

The authors declare that the research was conducted in the absence of any commercial or financial relationships that could be construed as a potential conflict of interest.

## Footnotes

^1^http://www.nature.com/scientificdata/

^2^See in particular Milham (2012) and Poldrack et al. (2013); http://openeuroscience.wordpress.com/; the collaborative interdisciplinary space and network www.neurobureau.org, for examples.

^3^http://fcon_1000.projects.nitrc.org/indi/adhd200

^4^http://brainmap.org

^5^http://www.oasis-brains.org/

^6^http://neurosynth.org

^7^http://humanconnectome.org/

^8^http://www.databrary.org

^9^http://humanconnectome.org/

^10^See the August, 2013 meeting of the Presidential Commission for the Study of Bioethical Issues (Brookshire, 2013).

^11^www.eyewire.org

^12^www.backyardbrains.com
